# X-ray physico-chemical imaging during activation of cobalt-based Fischer–Tropsch synthesis catalysts

**DOI:** 10.1098/rsta.2017.0057

**Published:** 2017-11-27

**Authors:** Andrew M. Beale, Simon D. M. Jacques, Marco Di Michiel, J. Frederick W. Mosselmans, Stephen W. T. Price, Pierre Senecal, Antonios Vamvakeros, James Paterson

**Affiliations:** 1 Department of Chemistry, UCL, 20 Gordon Street, London WC1H 0AJ, UK; 2 Research Complex at Harwell, Harwell Science and Innovation Campus, Rutherford Appleton Laboratory, Didcot, Oxon OX11 0FA, UK; 3 School of Materials, Manchester University, Oxford Road, Manchester M13 9PL, UK; 4 ESRF, BP 220, 38043 Grenoble, France; 5 Diamond Light Source, Harwell Science and Innovation Campus, Didcot, Oxon OX11 0DE, UK; 6 BP Chemicals, Conversion Technology Centre, HRTC-DL10 Saltend, Hedon, Hull HU12 8DS, UK

**Keywords:** physico-chemical, tomography, cobalt, Fischer–Tropsch, spectroscopy, scattering

## Abstract

The imaging of catalysts and other functional materials under reaction conditions has advanced significantly in recent years. The combination of the computed tomography (CT) approach with methods such as X-ray diffraction (XRD), X-ray fluorescence (XRF) and X-ray absorption near-edge spectroscopy (XANES) now enables local chemical and physical state information to be extracted from within the interiors of intact materials which are, by accident or design, inhomogeneous. In this work, we follow the phase evolution during the initial reduction step(s) to form Co metal, for Co-containing particles employed as Fischer–Tropsch synthesis (FTS) catalysts; firstly, working at small length scales (approx. micrometre spatial resolution), a combination of sample size and density allows for transmission of comparatively low energy signals enabling the recording of ‘multimodal’ tomography, i.e. simultaneous XRF–CT, XANES–CT and XRD–CT. Subsequently, we show high-energy XRD–CT can be employed to reveal extent of reduction and uniformity of crystallite size on millimetre-sized TiO_2_ trilobes. In both studies, the CoO phase is seen to persist or else evolve under particular operating conditions and we speculate as to why this is observed.

This article is part of a discussion meeting issue ‘Providing sustainable catalytic solutions for a rapidly changing world’.

## 1. Introduction

X-ray physico-chemical or chemical imaging is a term that is increasingly being used to describe the process by which X-ray beams interrogate samples, often intact, to reveal how the samples vary in terms of their physical and/or chemical constitution [1–11]. The spatially resolved signals obtained can reveal information that would otherwise be lost in bulk measurements. Such local signals are often simpler to interpret by virtue of containing fewer phases. This information allows us to understand how and why the samples perform the way they do in their intended application. Studying intact materials rather than idealized powders allows for behaviour under industrially relevant conditions to be observed. Furthermore, the background signal from *in situ* apparatus/cell can be readily separated. For structured heterogeneous catalysts where such methods have been regularly employed, it is possible to rationalize why they work or why they fail.

The energy of the incident X-ray beam for a µ-X-ray diffraction (XRD)–computed tomography (CT) measurement may be increased to suit the experiment, for example when thicker/more dense samples are used, whereas the sample size for μ-X-ray fluorescence (XRF)–CT imaging (and also μ-X-ray absorption near-edge spectroscopy (XANES)–CT) is limited by the energy of the fluorescent X-rays and the density/composition of the sample. The attenuation length of the material and sample environment at both the incident and fluorescent X-rays must, therefore, be considered more carefully when constructing a μ-XRF–CT measurement or a multimodal experiment than for a mono-modal μ-XRD–CT experiment. However, multimodal experiments have many benefits. μ-XRF–CT and in particular μ-XANES–CT both give information on the distribution of non-crystalline materials that cannot be detected by μ-XRD–CT. Simultaneous collection of data ensures each technique measures the sample under identical conditions, ensuring that there is no structural change between each dataset, and also increasing the speed. Further to this, the information measured by each technique can be combined during analysis, for example using the absorption data, from the transmitted beam intensity, to correct for the self-absorption of fluorescent X-rays. This is particularly beneficial when measuring more concentrated samples or if projections covering 180° of rotation are used, whereby a shadowing effect may be observed due to fluorescent X-rays from the far side of the sample being partially absorbed as they pass through the sample to reach the fluorescence detector.

In this work, we demonstrate the application of both µ-XRD–CT and multimodal tomography in an *in situ* reduction study (in H_2_) of Co-based catalysts used in low-temperature Fischer–Tropsch synthesis (FTS); a process with a significant history which continues to play an important role in global fuel production and, because it does not matter how or where the syn-gas it uses is derived, is likely to play an increasingly important role as part of a sustainable technology solution [12,13]. This work builds on some recent similar studies where we observed, using the same chemical tomography, the impact of spatial variation in the physical characteristics (i.e. phase and size) of the Co nano-crystallites during reduction and under FTS conditions; these observations have important implications for understanding the true nature of active Co species responsible for the formation of the desired C–C coupled reaction products. The results obtained here again illustrate the importance of this spatial information when developing structured catalysts for the FTS reaction.

## 2. Experimental set-up

### (a) Preparation of Co/SiO_2_ catalyst

Typically, 15 g of silica (Grace Davison, SG 432, 180–300 µm particle size, pore volume = 1.2 ml g^−1^) was dried in a fan oven at 100°C for 2 h. The dried support was impregnated at incipient wetness point with a solution of cobalt nitrate hexahydrate (Alfa Aesar, 98%) and perrhenic acid (Sigma Aldrich, 75–85 wt% in H_2_O). Co-deposition of Co and Re at the same time (before calcining) ensured thorough mixing and therefore uniform promotion. The impregnated support was calcined in a muffle furnace using the following programme: ramp at 2°C min^−1^ to 100°C and hold for 3 h, then ramp at 2°C min^−1^ to 300°C and hold for 5 h. Titanium isopropoxide (Alfa Aesar, 97%) in isopropanol (Fischer, 99.5%) was then deposited by incipient wetness. The sample was calcined following the same programme as in the previous step. The resulting catalyst had the composition 5% TiO_2_/10% Co 1% Re/SiO_2_, but will be referred to as Co/SiO_2_ throughout this paper.

### (b) Preparation of Co/TiO_2_ catalyst

A 1.5 mm trilobe comprising either 10 or 20 wt% Co/TiO_2_ catalyst was prepared by pore-volume impregnation of a TiO_2_ support (mixed anatase and rutile) using a solution of Co(NO_3_)_2_·6H_2_O (and 1 wt% equivalent of Mn in the form of Mn(NO_3_)_2_·6H_2_O) followed by room temperature drying and calcination at 300°C (ramp 5°C min^−1^ and dwell for 4 h).

### (c) Multimodal X-ray tomography of submillimetre Co/SiO_2_ catalyst particles

A single grain of Co/SiO_2_ was loaded into a 300 µm OD quartz capillary (10 µm wall thickness) and held in place using quartz wool. The capillary was mounted on top of a motorized gothic-arch bearing stage with XY travel to allow for centring of particles on the axis of rotation. Gas flow through the capillary was delivered via an inlet valve at the base of the capillary mount. Gas flow was 16 ml min^−1^ He for the room temperature measurement on the as-received (calcined) samples. Samples were reduced at 400°C under flowing 5%H_2_/He with a ramp rate 1°C min^−1^. The temperature was then reduced to 150°C at 10°C min^−1^ before the gas mix changed to 5% (3H_2_ : CO)/He, with a 16 ml min^−1^ flow rate. Finally, the temperature was increased to 220°C at 5°C min^−1^. These operating conditions were for methanation, due to the lower pressure, and the gas hourly space velocity was in excess of that used in the offline testing. Tomographic data were collected on the samples as received (i.e. calcined), in the active state (after reduction), and finally under methanation conditions.

Absorption–CT, XRD–CT and XRF–CT data were measured on beamline I18 at Diamond Light Source. Data were collected simultaneously using an incident X-ray energy of 13 keV. The sample was rastered across the beam in a translate-rotate data collection scheme, with an XRF spectrum and XRD pattern collected at 5 μm intervals with a collection time of 1 s per pixel. A total of 61 translation steps at each rotation angle and 50 rotations (in 3.6° steps) were used. Absorption data were collected by means of an ion chamber behind the sample, XRD images were recorded with a Photonic Sciences CMOS-based X-ray imaging detector and XRF spectra with a Vortex Si drift detector. The CMOS was calibrated using a LaB_6_, and data azimuthally integrated from two-dimensional images to one-dimensional patterns using Dawn v. 1.9 [14]. Reconstruction of the data was by filtered back-projection to create the final data volume.

XANES–CT data were collected in a similar manner to XRF–CT, and included the collection of the X-ray absorption signal through the particle to allow for self-absorption corrections. This was collected on the sample under methanation conditions, after the collection of the XRD–CT data. The X-ray beam energy was set to approximately 50 eV below the absorption edge of interest and the sample was rastered across it with a time per pixel of 0.1 s. Once the imaging of the row was completed, the sample was then rotated by 3.6° and the imaging repeated. The process was repeated 50 times until the particle had been rotated by 180° forming a sinogram with sufficient rotations to enable an accurate reconstruction. Four further sinograms were collected at 10 eV intervals approaching the absorption edge, at 1 eV intervals over the edge for 50 eV, at 2 eV intervals for a further 10 eV and, finally, at 10 eV intervals with the last sinogram collected at 165 eV past the absorption edge. A total of 80 sinograms were collected over the Re L_3_ absorption edge and 30 over the Co K absorption edge. Time limitations meant only the pre-edge region of the Co K edge XANES–CT was clearly defined—with a few points over the edge to allow for normalization. While the latter contained too few points to reconstruct a full XANES spectrum, it allowed for differences in the pre-edge features and position of the absorption edge to be compared; differences in intensity at 7709 eV are large, and while these cannot be definitively identified as Co^0^ or Co^2+^, because the whole edge shape is not present, the contrast is consistent with the XANES spectra in electronic supplementary material, figure S1, and also the phases identified by XRD–CT.

### (d) X-ray diffraction computed tomography of Co/TiO_2_ millimetre-sized trilobes

XRD–CT measurements were made at station ID15A of ESRF using a 93 keV monochromatic beam focused to have a spot size of 50 × 50 µm. Diffraction patterns were collected on a Perkin Elmer flat panel detector. For each tomographic time slice, diffraction was recorded at 70 translations spaced 50 µm across the body each with 60 rotations of 3°, corresponding to 4200 diffraction patterns. The acquisition time per point was 500 ms and each XRD–CT scan lasted approximately 45 min (inclusive stage movement and detector read-out time). Powder ring data were obtained and these were radially integrated (using datasqueeze software) after calibrating the detector and its response with an 8 nm CeO_2_ standard (http://www.datasqueezesoftware.com/index.html). For each observed intensity in these radially integrated patterns, a sinogram was constructed and then filtered back-projected to a 66 × 66 pixel image.

Catalyst reduction was performed in a 2.0 mm outer diameter (o.d.)/1.8 mm internal diameter (i.d.) glass reactor cell at atmospheric pressure and was performed under 10 ml min^−1^ of H_2_ from room temperature to 120°C with a ramp of 5°C min^−1^ and then under 25 ml min^−1^ of H_2_ from 80 to 310°C with a step at 150°C. The trilobe was placed in the middle of the quartz tube and quartz wool packed either side and in between the trilobe and the reactor cell wall in order to minimize reactant gas bypass. At the outlet to the reactor, a loose fitting ‘hood’ with gas extraction lines was placed over the outlet of the quartz cell, which prevents exposure to air via back flow. To maintain the correct (and a uniform) temperature at the sample, two heat guns were mounted on the translation stage, which move with the reactor, thereby maintaining a constant sample temperature [15].

The data presented in this work are plotted in terms of reflection area or else using scaled intensities when quantifying the amount of cobalt present (as a ratio) of a particular crystalline phase. Intensity data were obtained from reflection profiling (via fitting of the Bragg peak using a Gaussian function) of the Co_3_O_4_ (311), CoO (200) and face-centred cubic (fcc)-Co (111) phase from their respective crystallographic data using GSASII [16]. Subsequently, from the ratio of these intensities, the wt% of fcc-Co is plotted in, working on the basis that the total Co %age = CoO + Co + Co_3_O_4_. Based on the reproducibility of the data fitting process, we estimate the error on the quantities present to be ±3%.

## 3. Results and discussion

### (a) Single Co/SiO_2_ particle after calcination

**Figure 1.**
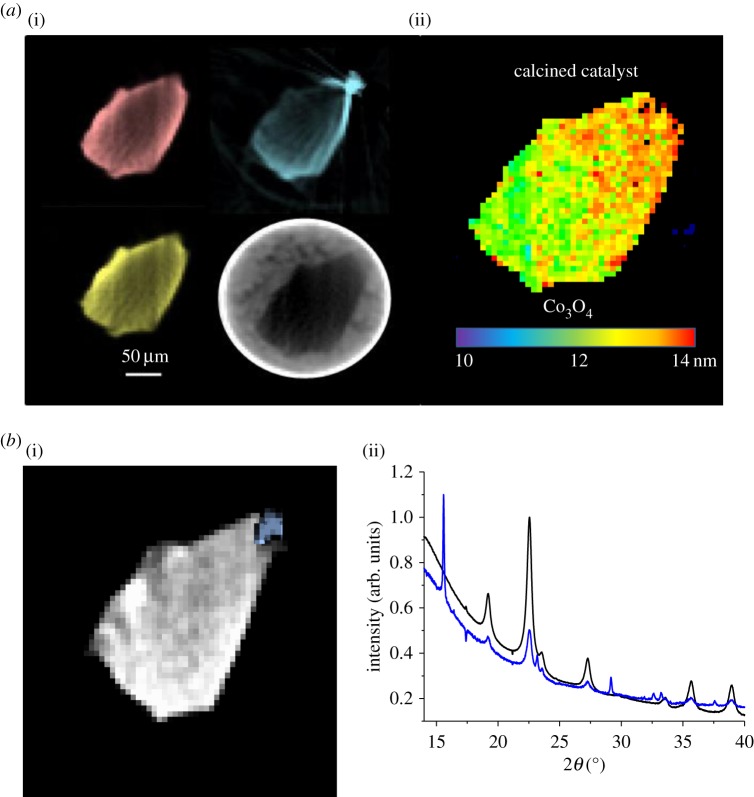
(*a*) XRF–CT and absorption–CT of Co/SiO_2_ catalyst after calcination. Left: Co XRF signal (upper left, red), Re XRF signal (lower left, yellow), Ti XRF signal (upper right, blue) and X-ray absorption (lower right, grey). Right: crystallite size distribution of Co_3_O_4_. Colour bar represents average crystallite size per pixel in nanometre. Each pixel represents a 5 × 5 μm region. (*b*) Left: Co/SiO_2_ sample XRD–CT reconstruction of peaks for anatase TiO_2_ (101 reflection, blue) and Co_3_O_4_ (311 reflection, white/grey). Right: corresponding spatial resolved diffraction patterns. Crystalline TiO_2_ is only located in the top right corner, corresponding to the highest Ti loading (cf. (*a*)). (Online version in colour.)

The elemental distributions of the catalyst and promoters in the calcined catalyst before reduction treatment are displayed in figure 1*a*. All three metals are concentrated at the edge of the SiO_2_ support, with some penetration (particularly Co and Re) to the core of the SiO_2_. The Ti distribution exhibits the greatest inhomogeneity, with a large concentration at the top of the particle; this was sufficient to cause some artefacts (apparent loss of signal next to the hot spot, and some small streaks radiating from it) in the reconstruction due to only measuring over 180° rotations, even after absorption corrections were applied. Corresponding μ-XRD–CT data shown in figure 1*b* reveal the Ti support modifier to be present almost entirely in non-crystalline form; the one exception being the region of highest concentration where some of the TiO_2_ anatase polymorph is present, as has been previously observed [1]. The Re promoter is not present in crystalline form; however, spot μ-XANES measurements confirm its presence in form very similar to that of the perrhenate complex (ReO_4_^−^) [1]. The distributions of Co and Re for both samples are highly correlated, which is expected because Co and Re are incorporated in the same step during preparation; indeed, it has been proposed that the co-deposition of Re and Co on the support results in the incorporation of Re into the Co nano-crystallite structure [17,18].

The Co is present entirely as Co_3_O_4_ on the SiO_2_ support; i.e. no evidence was found for any cobalt silicate (CoSi_2_O_4_) or cobalt titanate phases (CoTi_2_O_4_). The Co_3_O_4_ peaks are sufficiently well defined to allow for performing a Scherrer analysis in order to determine the average crystallite size per pixel; this was done by fitting the Co_3_O_4_ 311 peak with a pseudo-Voigt function revealing an average crystallite size of 12–13 nm (figure 1*a*). While previously we have observed the formation of some CoO in the presence of high concentrations of Ti in the form of anatase, the ‘hot spot’ of Ti present in the sample here appears to be a large anatase crystallite that is formed on the surface of the support, rather than a direct modification of the support and hence contains very little (if any) CoO [1].

### (b) Single Co/SiO_2_ particle after *in situ* reduction in H_2_

**Figure 2.**
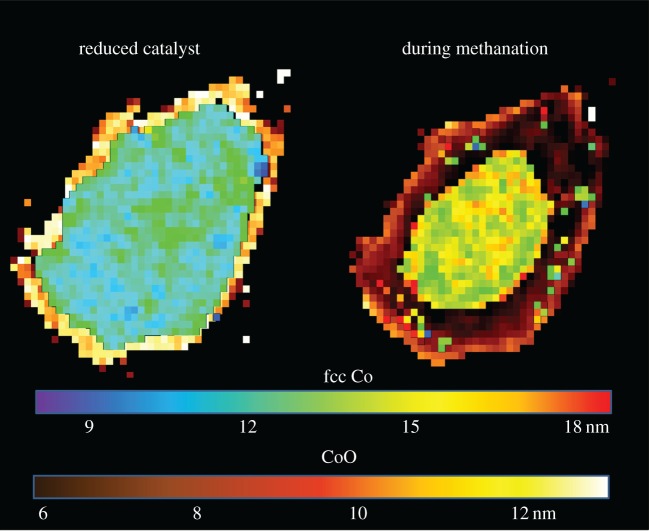
Crystallite size distribution of Co phases in the Co/SiO_2_ catalyst. Colour bars represent average crystallite size per pixel in nanometre. CoO (hot colour scheme, located at the edge of the support) and cubic Co (rainbow colour scheme, located in the core of the support). Each pixel is a 5 × 5 μm region. (Online version in colour.)

Following reduction treatment, the Co is reduced, predominantly to metallic Co; however, a thin shell of CoO remains present at the edge of the sample (figure 2). The metallic Co can be readily identified as fcc packed; however, Bragg reflections likely due to the presence of the hexagonal close packed (hcp) polymorph can also be seen; the very broad nature of Bragg reflections for this phase in addition to significant overlap with reflections for the fcc phase makes it difficult to extract useful additional information (i.e. crystallite size) from the hcp phase. However, in our previous work, we performed a more precise modelling of the nano-crystallite structure using the Debye scattering equation to reveal these nano-crystallites to be intergrown/stacking faulted (i.e. containing a mixture of cubic and hexagonal packed) Co phases and that for this particular type of catalyst, these intergrown domains are typically approximately 50% the size of the fcc domains [1]. Notwithstanding this, the average Co crystallite size remains similar after reduction, suggesting that only oxygen loss from the nanoparticle took place (considering the differences in density and assuming the Co_3_O_4_ crystallites to be initially cuboidal and the fcc Co nano-crystallites to be spherical). While it is known that smaller crystallites (less than 7 nm) are susceptible to water-induced re-oxidation, there was no such spatial variation in Co crystallite size in the calcined sample (i.e. very few crystallites observed less than 10 nm), and so it is unlikely that this is the (sole) cause of the CoO formation at the particle edge [1,15,19,20]. More likely, the cause of this oxidation is the presence of trace levels of O_2_/H_2_O in the reactor, although it is only able to permeate approximately 15 µm into the sample. We note however that when measuring a packed bed that this effect was not seen [1].

### (c) Single Co/SiO_2_ particle studied *in situ* during methanation

The switch to ‘methanation’ conditions (CO + 3H_2_ → CH_4_ + H_2_O) saw a small increase in metallic crystallite size, particularly 150 µm in from the edges of the sample. Re-oxidation of the smallest Co nano-crystallites would cause the average metallic crystallite size to increase; however, it is also likely that some sintering is occurring because the Co is known to be mobile on SiO_2_, although there is some evidence that it can also be mobile on TiO_2_ [21,22]. As the methanation temperature is much lower than that for the reduction step, it is unlikely that the crystallite growth in this instance is temperature induced.

**Figure 3.**
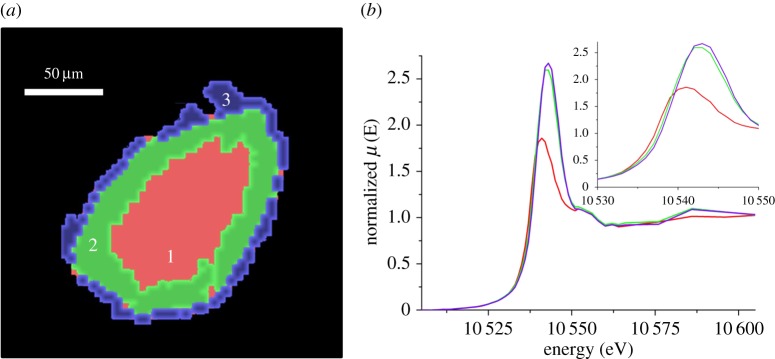
Cluster map of reconstructed Re L_3_ edge XANES–CT datastack of the inverse catalyst (left) and associated XANES spectra (right); cluster 1 (red), cluster 2 (green) and cluster 3 (purple). The inset (right) shows a close-up of change in edge position. Note the protrusion at the top of cluster 3 is an artefact from the reconstruction process coupled with the cluster analysis because no Re is present there in the XRF–CT reconstructions (cf. electronic supplementary material, figure S5). (Online version in colour.)

A comparison of the μ-XRD–CT with the μ-XANES–CT highlights the size sensitivity of each technique (electronic supplementary material, figure S2). Under methanation conditions, the metallic Co region, identified from the fcc Co 220 reflection, begins approximately 30 µm from the edge of the support, the outer layer being CoO. There is very little overlap in location between the two phases. On the other hand, the oxide shell identified from the μ-XANES–CT (figure 3) is nearer 50 µm thick, and the metallic core far smaller. As XANES does not require long-range order, this intermediate region between 25 and 50 µm from the edge of the particle is a region where non-crystalline CoO is being formed; most likely as a thin surface shell on metallic nano-crystallites as its surface is oxidized by the H_2_O generated by the methane formation.

The Co K edge XANES of Co_3_O_4_ and CoO possess strong absorption features at 20.1 and 17 eV above the absorption edge (7709 eV), respectively (dominated by 1 s–4p dipole transitions), whereas metallic Co possesses a shoulder in the absorption edge at +2.5 eV. The XANES spectrum of the calcined catalyst is fully consistent with Co_3_O_4_ (see electronic supplementary material, figure S2). Following the reduction treatment, the XANES shape does not resemble that of metallic Co. There is a significant shift in the energy of the absorption edge (consistent with reduction) yet it maintains a large peak at 7726 eV, consistent with CoO. This was the case after an extended reduction treatment (additional 2 h at 420°C). Previous bulk studies on the reduction in Co-based FT catalysts have reported between 10% Co up to 60% remains as oxide after reduction treatment; however, the use of promoters varied between studies [23]. In the cases of Re promotion, some residual CoO is still observed in XANES, despite the promoter facilitating the CoO reduction. The absorption edge peak increases in intensity once the catalysts are under methanation conditions, indicating an increase in the amount of CoO present. The re-oxidation of Co has been observed in previous bulk studies, even though it is not thermodynamically favourable and has been attributed to the size of the Co cluster, preferential oxidation of the surface of Co clusters, and also interaction with Re_2_O_7_. In this case, it is also highly likely that re-oxidation is caused by the H_2_O generated by the methanation conditions.

Re is present as a promoter to lower the reduction temperature of the Co via H_2_ spillover. To do so, the Re must first be reduced to its metallic state. XANES–CT spatially reveals a metallic Re core and a highly oxidized Re shell (figure 3). There is a direct correlation between the locations of oxidized Re and oxidized Co, and likewise for the metallic components. Re is incorporated within the Co nano-crystallites with direct Re–Co bonding having been previously observed by EXAFS [17,18]. The co-location in the XANES–CT of the metallic and oxidic regions of Re and Co is in agreement with this; however, the metallic Re component occupies a larger volume of the core of the particle than the metallic Co. There are two likely causes; first, Co is more susceptible to re-oxidation under methanation conditions; secondly, there may be a large proportion of the Re subsurface, because the re-oxidation begins on the surface of the nano-crystallites [24].

### (d) Effect of cobalt loading on the reducibility of Co/TiO_2_ trilobe catalysts

**Figure 4.**
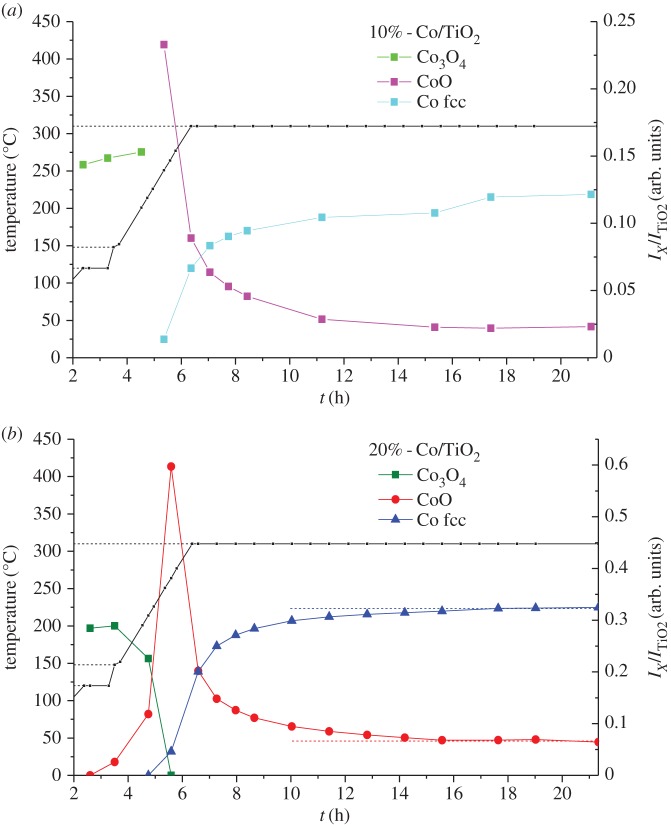
Composition profile compiled from the integrated and scaled reflection intensities for the cobalt phases (Co_3_O_4_ (311), CoO (200), fcc-Co (111)) from the summed two-dimensional diffraction data observed throughout the H_2_ reduction process and during the reduction of 10 (left) and 20 (right) % Co/TiO_2_, respectively. (Online version in colour.)

Co/TiO_2_ trilobes containing (10% wt and 20% wt of Co, respectively) promoted with a low (approx. 1%) amount of additional manganese have been characterized with XRD–CT during reduction. The data shown in figure 4 represent all of the summed µ-XRD–CT data recorded from the two-dimensional cross-section of the trilobe for both samples, acquired before and after reduction in H_2_. These data are plotted as scaled intensities from reflection profiling (via fitting of the Bragg peak using a Gaussian function) of the Co_3_O_4_ (311), CoO (200) and fcc-Co (111) phase from their respective crystallographic data using GSASII and therefore comprise a ratio of a particular crystalline phase [15,16]. At the beginning of the *in situ* experiment and as expected, Co_3_O_4_ obtained after calcination is the only cobalt-containing phase present. For the Co 10% wt sample, Co_3_O_4_ reduction begins when temperatures reach approximately 250°C with further reduction to the metallic form beginning at temperatures some approximately 20°C higher. For the 20% wt sample, reduction in Co_3_O_4_ begins already at temperatures as low as approximately 150°C, although complete conversion to CoO is not complete until the temperature reaches approximately 250°C, which is also the temperature at which CoO begins to reduce. From 18 h onwards until the conclusion of the reduction treatment (at 310°C), the reduced sample is dominated by contributions ascribable to fcc Co and in the case of the sample loaded with 20% Co, a small amount of unreduced CoO (approx. 20%); this perfunctory analysis reveals that, in this instance, the fcc Co phase to appear to be the majority phase present. We note that a small amount of hcp-Co (100 reflection) is also observed as a weak shoulder on the 111 reflection of TiO_2_ rutile. This significant overlap prevents a robust and meaningful analysis of the nature and properties of this Co phase. Furthermore, because the shoulder is weak, it suggests that it is not present in significant amounts (less than 10%) and, as such, no further analysis of this Co polymorph was performed.

**Figure 5.**
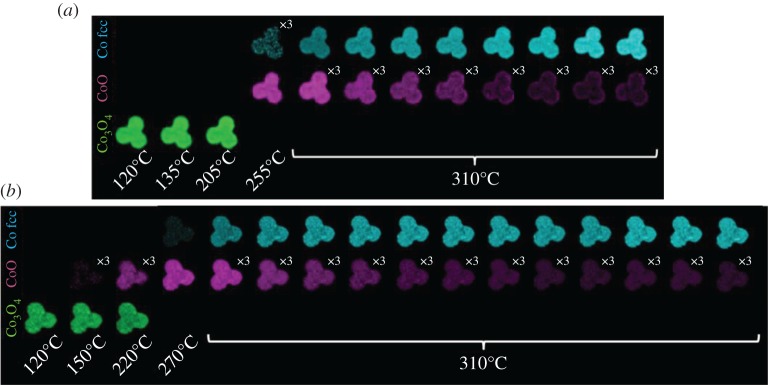
Reconstructed two-dimensional integrated reflection intensity maps for Co_3_O_4_ (220), CoO (200) and fcc Co (111) observed during reduction in (*a*) 10 wt % Co and (*b*) 20 wt % Co, respectively. Since the acquisition of the two-dimensional data took approximately 60 min, there are 11 and 16 observation steps as the sample is reduced, respectively. Multipliers depicted in the right-hand corner of some of the intensity maps (particularly CoO) indicate how the integrated intensities have been multiplied to enable plotting on a common axis. Note the brighter the pixel, the more that a particular phase is present.

The spatial dependency of the phase evolution presented above was determined from the CT-reconstructed two-dimensional intensity distribution maps which are shown in figure 5. Starting with the initial Co distribution, based on the intensity of the 220 reflection of Co_3_O_4_, we observed the distribution of Co to be very uniform irrespective of the loading; the edge of the right-hand lobe in the 10% wt (figure 5*a*) hints at a more egg-shell distribution in this part of the sample [25]. The data shown in figure 5 confirm the same changes reported in figure 4 for both samples, but in particular show that the Co distribution is retained irrespective of Co phase as the Co_3_O_4_ → CoO → fcc-Co metal phase transformation occurs with time/temperature. For the 10% wt sample, there is a clear egg-shell distribution for the ‘remaining’ CoO phase which can be seen to evolve during reduction (see electronic supplementary material, figure S3). There is no corresponding equivalent or inverted distribution for the metallic Co phase. From the reflection intensity analysis at the end of the reduction process, more than 90% of the cobalt in the middle of the sample is reduced to fcc-Co while in the outer approximately 150 µm, this amounts to 60%. By contrast, there is no such spatial variation seen in the 20% wt sample; however, there is a greater amount of evenly distributed ‘remaining’ CoO (20%).

Shown in electronic supplementary material, figure S4, are the two-dimensional maps of the intense Bragg reflections pertaining to the anatase and rutile phases set against the distribution of Co and CoO on the conclusion of reduction. No other Ti-containing phases (i.e. CoTiO_3_) were observed at any point in the experiment. Furthermore, there is clearly no obvious effect of TiO_2_ polymorph on the evolving Co phase distribution seen in the 10% wt sample.

### (e) Co/TiO_2_ crystallite size

**Figure 6.**
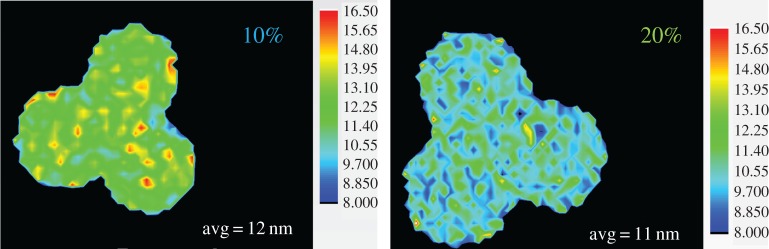
Reconstructed images of crystallite sizes (nanometres) of cobalt fcc at 310°C under H_2_ of the two TiO_2_ supported Co containing trilobes. The thermal scale bar on the right-hand side of each image indicates the correlation between pixel colour and Co crystallite size. The mean size (avg. value) is quoted at the bottom of each figure. (Online version in colour.)

Figure 6 contains maps of the nano-crystallite size for the tomographic slices of metallic fcc-Co at the end of the reduction as derived from the use of the Scherrer equation after profiling the 111 reflection. The mean crystallite size of fcc-Co is very similar in both samples at around 11–12 nm. The range of crystallites observed in both samples is also similar with minimum sizes ranging from approximately 8 to a maximum of 17 nm. However, it is clear that in both samples, but in particular in the 20% sample, that the majority of crystallites (modal value) have a size similar to the mean value in both cases. Furthermore, the distribution of these crystallites can be considered to be largely homogeneous across both samples. Shown in electronic supplementary material, figure S5, is a summed diffraction pattern of the two-dimensional data at the end of the reduction process and which have been normalized against the Bragg reflection (highest) intensity for the anatase 101 reflection. Besides the expected intensity difference due to the greater amount of Co in the 20 wt% sample, it is clear that the 111 reflection in both samples is very similar in shape and this is particularly noticeable when the intensities are normalized.

## 4. Summary and discussion

In our previous study of the Co/SiO_2_ catalyst, we also observed the various Co phases to be the only crystalline part of the catalyst particle; the SiO_2_ support and promoters Re and Ti being essentially amorphous. The distribution of these elements is generally biased towards the sample edge. However, the initial Co_3_O_4_ crystallite size is very uniform over the catalyst. During H_2_ reduction, the initial spinel phase is observed to reduce to yield CoO and eventually fcc Co plus an intergrown phase; this intergrown domain size is however notably smaller than the fcc-Co phase [1]. The Re is incorporated into the Co nano-crystallite, and has an initial oxidation state consistent with Re^4+^. At the end of the reduction, a 15 µm shell of CoO was observed surrounding an fcc Co core of the particle; this incomplete reduction is unexpected and might be a consequence of trace amounts of O_2_ or H_2_O in the feed gas. Alternatively, it might indicate a moving waterfront from the centre to the edge of the catalyst and in which case, a longer reduction time might eventually lead to complete reduction. The Re exhibits a very similar behaviour to that of Co, i.e. it has been reduced to Re^0^ in the core of the particle, but at the edges maintains a similar XANES profile to Re^4+^ where CoO is also present. On switchover to methanation conditions, re-oxidation of small Co nano-crystallites occurs, particularly at the sample periphery, as has been observed previously; this phenomenon arising as a consequence of water produced as a co-product of the catalytic process.

For the Co/TiO_2_ trilobes, the lower temperature for the onset and the greater extent of reduction in the 20% wt sample than for the 10% wt sample suggested the presence of a reduced metal–support interaction in the sample with higher loadings. We propose that the remaining CoO present exhibits an intimate interaction with TiO_2_ and which requires a higher reduction temperature/longer reduction time to form metallic Co. However, this increase in temperature/time is not expected to be all that higher/longer because most of the Co is present as metallic Co already. The egg-shell CoO distribution (approx. 150 µm in from the sample edge) seen in the 10% wt sample is more difficult to rationalize; as with the single particle study, this could be due to the presence of trace amounts of O_2_ or H_2_O in the inlet gas which are difficult to remove completely. Alternatively, because this phenomenon was already seen for the single particle containing a similar Co loading, this may be a consequence of the reduction process with the outer parts of the particle seeing more water than the centre. The fact that this distribution is not seen in the 20% wt Co sample suggests that it is the Co in contact with the TiO_2_ that is difficult to reduce and that these Co crystallites are likely to be small; this same phenomenon having been previously observed for small (less than 7.5 nm) Co nano-crystallites on γ-Al_2_O_3_. For both Co loadings, the average nano-crystallite size and the spatial distribution is very similar, suggesting a robust method has been developed for controlling Co nano-crystallite size irrespective of loading.

## 5. Conclusion

The chemical imaging techniques demonstrated here clearly have much potential for revealing a more detailed understanding of the correlation between structure and function in structured materials in catalysis and beyond. In this particular study, it is clear that the information provided allows for a greater appreciation of the impact of catalyst formulation and potentially how a processing step (pre-reduction before use in reaction) affects the structure of a heterogeneous catalyst, how this affects final performance and how these parameters could be optimized using such methods. The time resolution in which these imaging experiments can be performed is already sufficiently fast (seconds to minutes) to examine solid-state changes in two dimension as they happen but with continuously improving source brightness and detector performance we observe that already there are reports of time-resolved three-dimensional imaging (a term we and others have recently described as five-dimensional imaging in which the fourth and fifth dimensions are time and spectral/scattering information, respectively) [26,27]. We note however that with all of the experimental advancements being made (improvements in synchrotron sources, detectors, environmental cells), it is crucial that the associated processing and analysis techniques and software are developed to keep pace with the size and quality of data being collected. While packages are being developed for tomographic data reconstruction (e.g. MMX-I, TomoPy), not all are suitable for the reconstruction of XRD–CT due to the additional data dimension [28,29]. Savu, developed by Diamond Light Source, is designed with these multimodal data in mind, and EasyDD is also suitable for processing XRD–CT [30–32]. As well as processing and reconstructing the data in a practical timescale (i.e. during the beamtime to enable visualization of the data), these methods can also apply filters to the data, separating out the signal from polycrystalline diffraction rings and single crystal spots, thereby aiding the visualization and interpretation of the data. With these developments already underway, these imaging approaches are fast becoming sufficiently user-friendly and more widely available rendering these exciting times in our attempts to more fully interrogate the impact of micro-scale chemistry on macro-scale objects in catalysis and beyond.

## Supplementary Material

Supplementary Information

## Data accessibility

Processed datasets can be accessed via the following datalink: http://tiny.cc/PTA_RS_JUNE2017.
