# Supplementary material for: X-ray physico-chemical imaging during activation of cobalt-based Fischer–Tropsch synthesis catalysts
Source: Philos Trans A Math Phys Eng Sci. 2017 Nov 27;376(2110):20170057. doi: 10.1098/rsta.2017.0057 (PMC5719219; doi:10.1098/rsta.2017.0057)
Supplement: Supplementary Information [file rsta20170057supp1.docx]

Electronic Supplementary Information for:

X-ray Physicochemical Imaging during activation of Cobalt-based Fischer-Tropsch Synthesis Catalysts

by

Andrew M. Beale*^a,b^, Simon D. M. Jacques^b,c^, Marco Di Michiel^d^, J Frederick W. Mosselmans^e^, Stephen W. T. Price^e^, Pierre Senecal^a,b^, and Antonios Vamvakeros^a,b^, James Paterson^f^

*a Department of Chemistry, UCL, 20 Gordon Street, London, WC1H 0AJ, UK*

*b Research Complex at Harwell, Harwell Science and Innovation Campus, Rutherford Appleton Laboratory, Didcot, Oxon, OX11 0FA, UK*

*c School of Materials, Manchester University, Oxford Road, Manchester, M13 9PL, UK*

*d ESRF, BP 220, F-38043 Grenoble, France*

*e Diamond Light Source, Harwell Science and Innovation Campus, Didcot, Oxon, OX11 0DE, UK*

*^f^BP Chemicals, Conversion Technology Centre, HRTC-DL10 Saltend, Hedon, Hull, HU12 8DS, UK*

*Author for correspondence ([Andrew.beale@ucl.ac.uk](mailto:Andrew.beale@ucl.ac.uk)).

† Department of Chemistry, UCL, 20 Gordon Street, London, WC1H 0AJ, UK & Research Complex at Harwell, Harwell Science and Innovation Campus, Rutherford Appleton Laboratory, Didcot, Oxon, OX11 0FA, UK

| 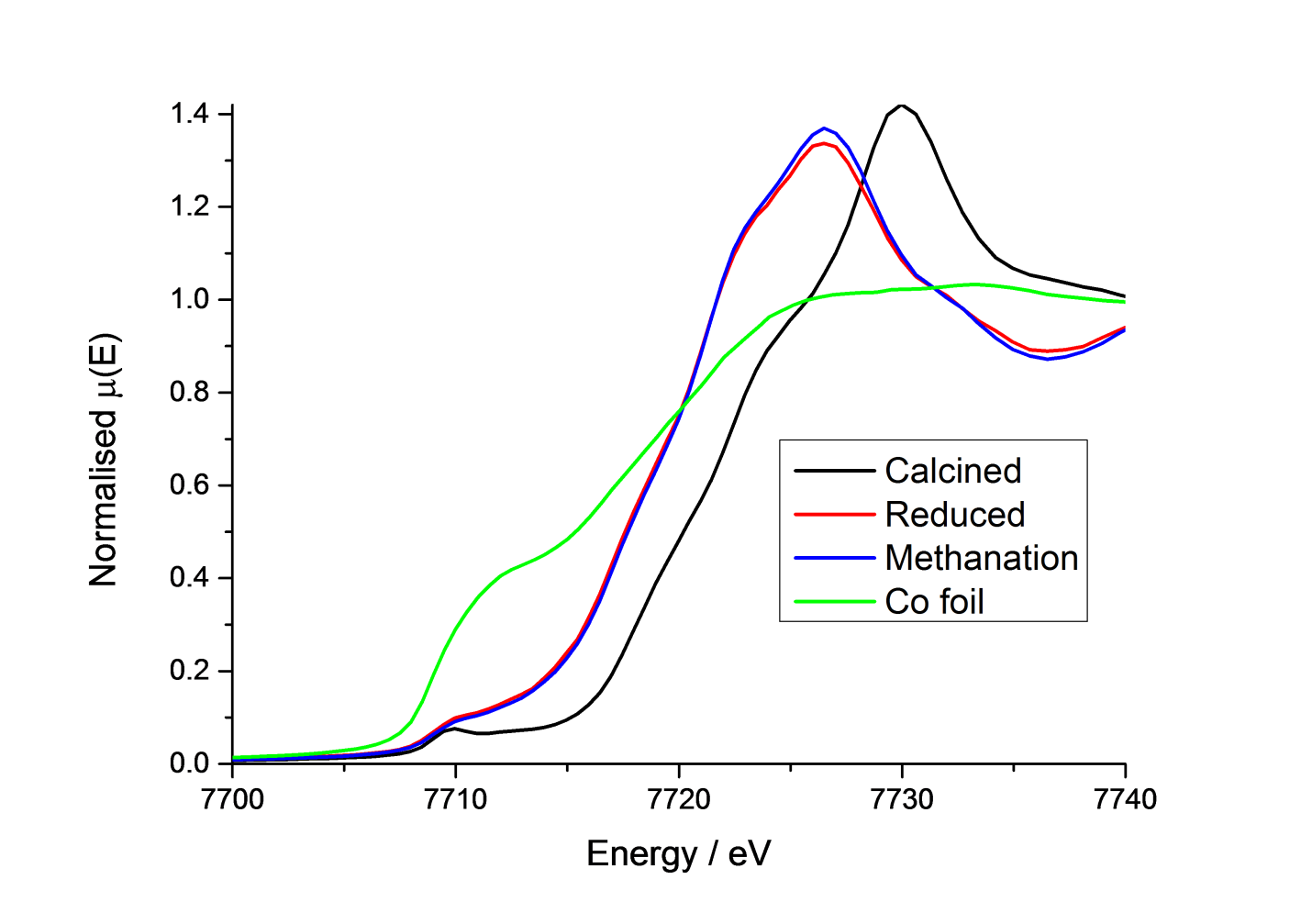 |
| --- |
| SI Figure 1: Co/SiO_2_ XANES. Calcined Profile fully consistent with Co_3_O_4_. XANES profiles after reduction and during methanation consistent with a mixture of metallic Co and CoO. |

| 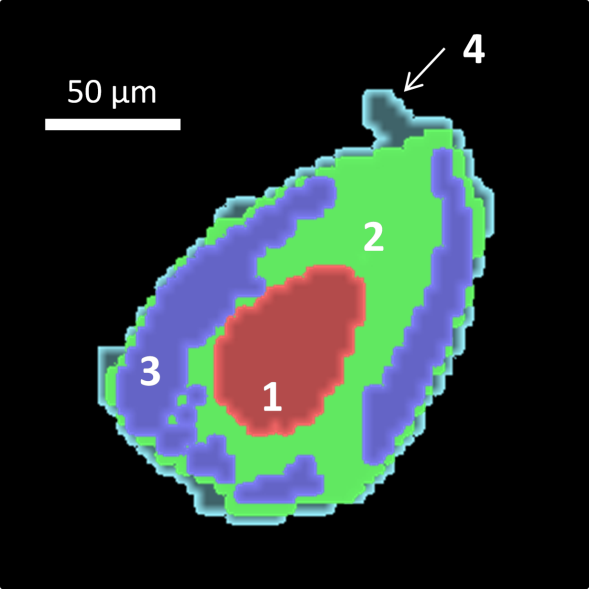 | 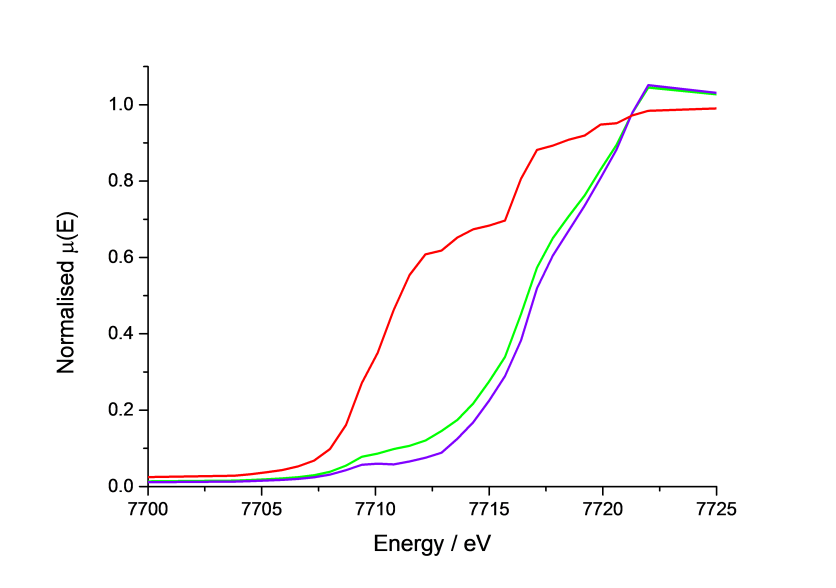 |
| --- | --- |
| SI Figure 2: XANES-CT of Co/SiO_2_ catalyst during methanation conditions (left) associated XANES spectra (right) ; cluster 1 (red), cluster 2 (green), cluster 3 (purple).  The 4th cluster on the map has a negligible intensity XANES spectrum, and is an artefact from the reconstruction process. The profile of the the pre-edge and relative height of the absorption at 7709 eV are consistent with metallic Co (cluster 1) and oxidised Co (clusters 2 and 3). | |

| 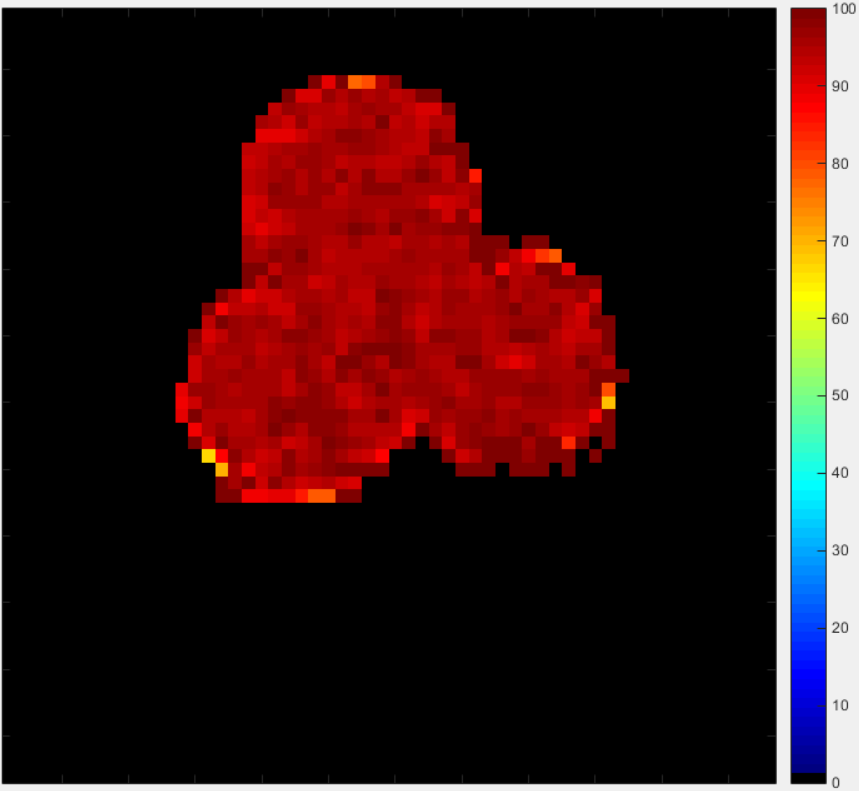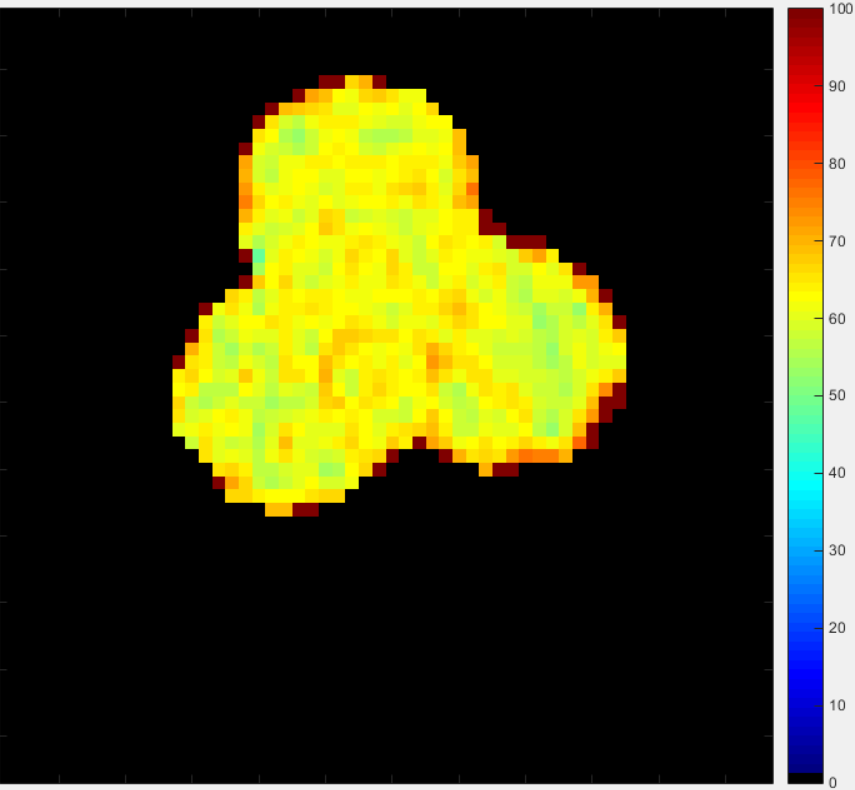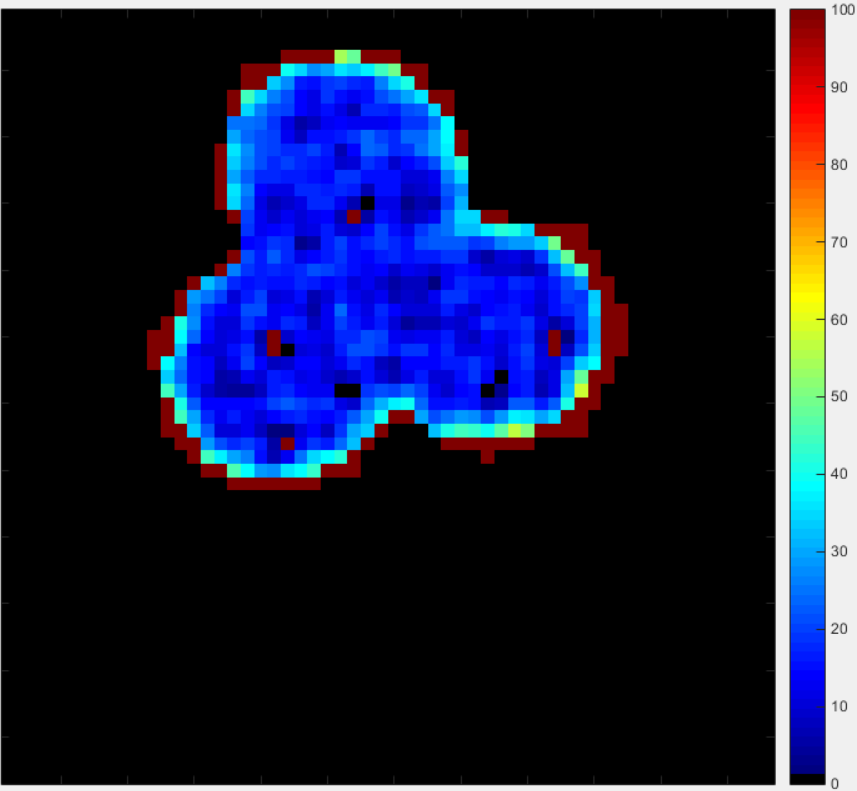 CoO  Cofcc 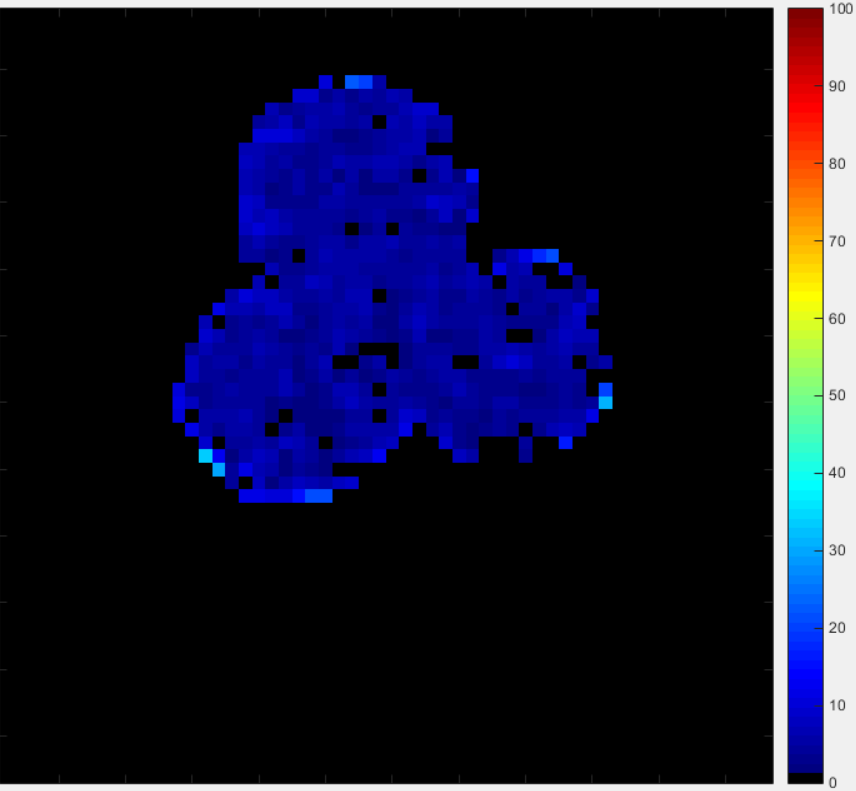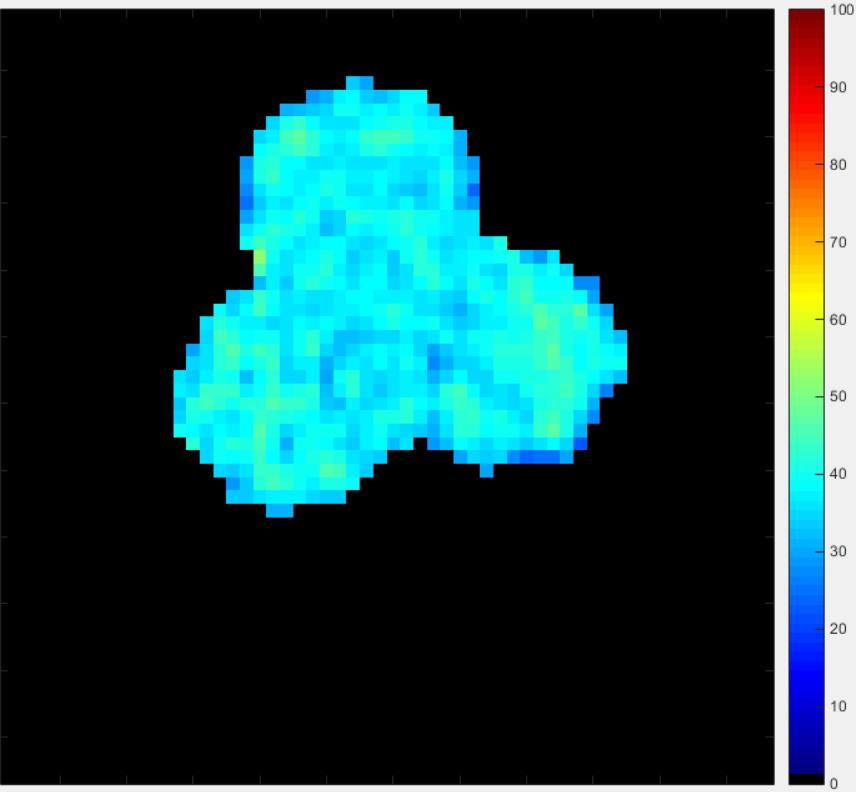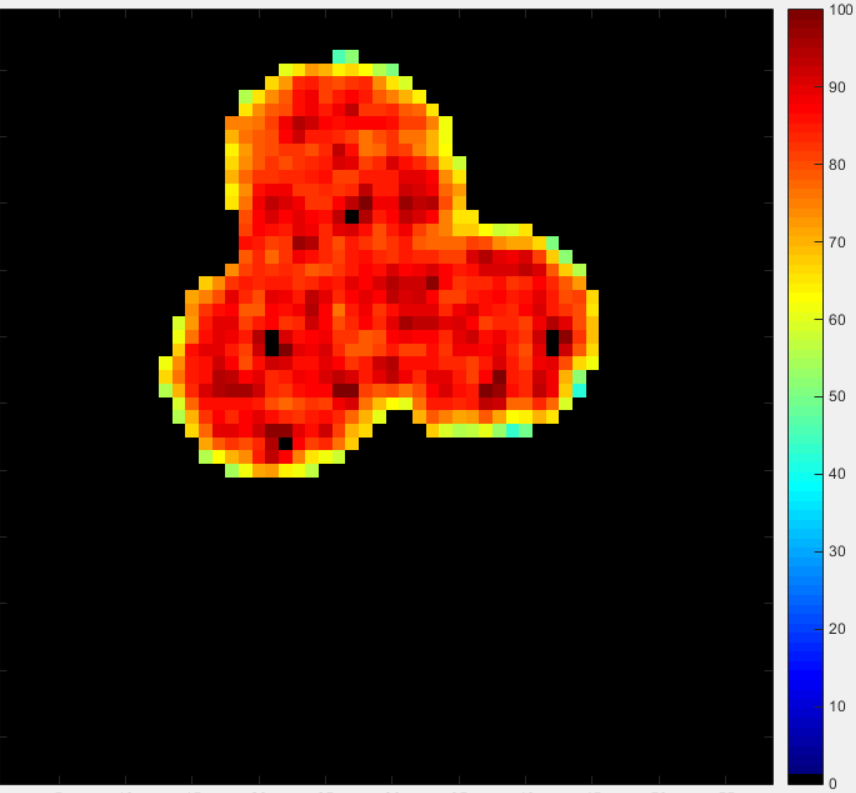 End of reduction (310°C)  250°C  Mid reduction (310°C) 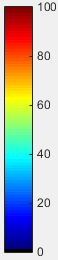 |
| --- |
| SI Figure 3: Reconstructed percentage composition images for the cobalt phases CoO and fcc Co during reduction for the 10 wt. % Co catalyst. |

|  | **Anatase** | **Rutile** |
| --- | --- | --- |
| 10 wt. % |  |  |
| 20 wt. % |  |  |

SI Figure 4: Reconstructed images of titanium and cobalt phases observed at the end of the reduction process of two TiO_2_ supported Co catalyst.

| 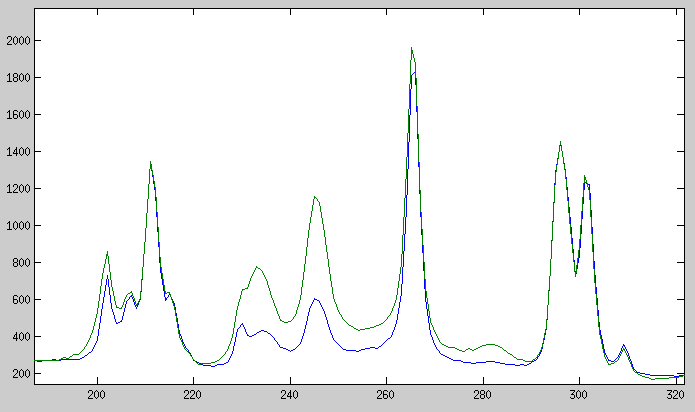 **10%Co/(Mn)TiO_2_**  **20%Co/(Mn)TiO_2_** | 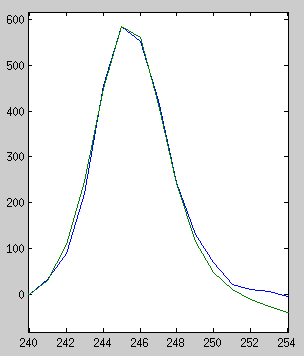 Normalised |
| --- | --- |
| SI Figure 5: Diffractograms at 310 °C under H_2_ of the two TiO_2_ supported Co catalyst with a normalisation (right) of the most intense (111) Co fcc reflection spanning the detector channels between 240 and 250. Note the similarity of the peak shape in the left image which confirms the close similarity of the average fcc Co particle size seen in both samples. | |

Processed datasets can be accessed via the following datalink: http://tiny.cc/PTA_RS_JUNE2017
